# Genomic Analysis of Chilean Strains of* Campylobacter jejuni* from Human Faeces

**DOI:** 10.1155/2019/1902732

**Published:** 2019-07-08

**Authors:** Arturo Levican, Ignacio Ramos-Tapia, Isabel Briceño, Francisco Guerra, Benjamin Mena, Carmen Varela, Lorena Porte

**Affiliations:** ^1^Tecnología Médica, Facultad de Ciencias, Pontificia Universidad Católica de Valparaíso, Avenida Universidad 330, 2373223 Valparaíso, Chile; ^2^Center for Bioinformatics and Integrative Biology (CBIB), Facultad de Ciencias de la Vida, Universidad Andres Bello, Avenida Republica 330, Santiago, Chile; ^3^Laboratorio Clínico, Hospital Naval Almirante Nef, Viña del Mar, Chile; ^4^Laboratorio Clínico, Clínica Alemana de Santiago, Facultad de Medicina Clínica Alemana, Universidad del Desarrollo, Santiago, Chile

## Abstract

*Campylobacter *spp., especially* C. jejuni*, are recognized worldwide as the bacterial species that most commonly cause food-related diarrhea.* C. jejuni* possesses many different virulence factors, has the ability to survive in different reservoirs, and has shown among isolates the emergence of Antimicrobial Resistance (AMR). Genome association analyses of this bacterial pathogen have contributed to a better understanding of its pathogenic and AMR associated determinants. However, the epidemiological information of these bacteria in Latin American countries is scarce and no genomic information is available in public databases from isolates in these countries. Considering this, the present study is aimed to describe the genomic traits from representative* Campylobacter* spp. strains recovered from faecal samples of patients with acute diarrhoea from Valparaíso, Chile.* Campylobacter* spp. was detected from the faeces of 28 (8%) out of 350 patients with acute diarrhoea, mainly from young adults and children, and 26 (93%) of the isolates corresponded to* C. jejuni*. 63% of the isolates were resistant to ciprofloxacin, 25.9% to tetracycline, and 3.5% to erythromycin. Three isolates were selected for WGS on the basis of their* flaA*-RFLP genotype. They belonged to the multilocus sequence typing (MLST) clonal clomplex (CC) 21(PUCV-1), CC-48 (PUCV-3), and CC-353 (PUCV-2) and presented several putative virulence genes, including the Type IV and Type VI Secretion Systems, as well as AMR-associated genes in agreement with their susceptibility pattern. On the basis of the wgMLST, they were linked to strains from poultry and ruminants. These are the first genomes of Chilean* C. jejuni* isolates available in public databases and they provide relevant information about the* C. jejuni* isolates associated with human infection in this country.

## 1. Introduction

*Campylobacter* spp., especially* Campylobacter jejuni *and* Campylobacter coli*, have become important for public health and are recognized worldwide as the bacterial species that most commonly cause food-related diarrhoea [1, 2]. The incidence rate of campylobacteriosis in the United States had been estimated as 14.3 per 100,000 in 2012, while in Europe, 246,307 cases were reported during 2016 according to EFSA [3–5].* Campylobacter* species produce generally self-limiting gastroenteritis; some complications such as septicaemia, meningitis, haemolytic-uremic syndrome, pancreatitis, and abortions have been reported. In addition, postinfection sequelae such as reactive arthritis and Guillain-Barre syndrome (GBS) have been attributed to these species [5, 6] (Moore et al., 2005; Willison et al., 2016). In this line, some authors have estimated that the risk of GBS after* C. jejuni* infection ranges between 1 in 1058 and 1 in 5000 cases of campylobacteriosis, depending on the serotype involved [7, 8]. Furthermore, evidence has been raised on the association between symptomatic and asymptomatic infections of* Campylobacter *and growth reduction among Peruvian children, where magnitude of growth retardations correlates with severity of infection [9]. Despite this, the epidemiological importance of all* Campylobacter* spp. in Latin American countries is overall underestimated and these bacteria are not considered of major public health importance. This is mainly because clinical laboratories do not search for* Campylobacter* routinely due to their limitations to fulfill the technical requirements for its isolation. Two independent studies carried out in Concepción, Chile, and Santiago, Chile [10, 11], have reported incidences of 2.7% and 6.1% among patients with diarrhoea, respectively. Those authors suggested that selective culturing for* Campylobacter* should be included in the routine bacteriological stool workup.

*C. jejuni* is the most frequently isolated species among patients with diarrhoea (about 90%), followed by* C. coli*, with 5-10% of cases [12]. For that reason,* C. jejuni* is the most studied species. In this regard, although the association between virulence factors and pathogenicity is not completely proven, several putative virulence factors have been observed in this species. For instance, it is able to perform N-glycosylation of over 30 related proteins related with colonization and invasion of intestinal cells [13]. Furthermore, its flagellum can be used for movement and also to secrete invasive antigens such as the* Campylobacter* Invasive Antigens (*Cia*), and it is able to control the cell cycle of intestinal cells through the Cytotoxic Distending Toxin (*cdt* gene subunits A, B, and C) [13]. Moreover, other genes associated with cell adhesion and invasion such as* virB11*,* icbA*,* pldA, wlaN, iamA, cgt*, and* cadF* have been identified [14, 15].

*Campylobacter *spp. infections are self-limiting; therefore, the antibiotic treatment is not recommended unless these patients experience fever and bloody or persistent diarrhoea [16]. In those cases, the WHO does not recommend the use of empirical therapies for* Campylobacter* spp. due to the emergence of resistance to fluoroquinolones, tetracycline and even erythromycin, which are the treatment of choice [17, 18]. Moreover, the susceptibility to antibiotics has been associated with the presence of virulence traits. For instance, Lapierre et al. [19] observed a wide distribution of putative virulence genes associated with adhesion, invasion, and cytotoxicity in Chilean* Campylobacter* isolates originated from foods of animal as well as from faecal samples obtained from animals. Moreover, a relationship between the presence of putative virulence genes and their susceptibility to antibiotics was observed [19].Therefore, the surveillance for antimicrobial resistance is necessary in these bacteria; however, as commented before, in Chile neither routine searches for these bacteria in stools of patients with diarrhoea nor their surveillance for antimicrobial resistance is so far being carried out.

Quinolone resistance in* Campylobacter *has been associated with single nucleotide mutations at the level of their target (*gyrA*), especially the Thr86Ile* GyrA* which is the most common mutation among clinical and veterinary isolates, but also to the presence of efflux pumps [16, 20]. In the case of tetracycline, the gene* tetO *induces a conformational change in the ribosome that causes the release of the antibiotic [20]. The high levels of resistance to antibiotics have been attributed to their use as growth stimulators in different species of animals in livestock [20]. A recent study evidenced a high resistance to ciprofloxacin (48 % of isolates) among strains of* C. jejuni* isolated from faecal samples of people with acute gastroenteritis in central and southern Chile between 2006 and 2015 [21]. The authors observed that most of ciprofloxacin-resistant strains were grouped into three dominant MLST clonal complexes (CC-21, CC-48, and CC-353), while only one of the strains belonging to CC 45 was resistant to erythromycin. The authors suggested that there is a dissemination of the resistant clonal lineages, which are responsible for the cases of campylobacteriosis [21].

Considering that the current understanding of the pathogenic determinants of* Campylobacter* is limited, recent studies have demonstrated the utility of Genome Wide Association Studies (GWAS) to the study of such determinants in this pathogen [22–24]. This is a top down approach for molecular markers discovery by comparing the genomic content of test and control strains (Buchanan et al., 2017). In a recent study, Buchanan et al. [24] applied a GWAS to identify genetic determinants among* C. jejuni* lineages associated with human disease in Canada. A total of 25 genes were identified which could be used as diagnostic markers for molecular-based assessment, while only four of them used in combination were able to detect 90% of the clinical associated isolates. Some of these genes have a role in important metabolic processes and others have unknown function but were associated with catalysis and transport [24]. Other studies have applied this approach to determine the genetic traits indicative of pathogenicity and zoonotic potential of* Campylobacter* spp. [22]. The analysis uncovered 2 groups of genomes, one associated with crows and another called “generalist.” This latter group included isolates from multiple host species including those implicated in human disease and were associated with the presence of potential virulence traits and antibiotic resistance [22]. However, previous studies do not include Chilean isolates, as no genomic information is available in public databases from isolates of pathogenic* Campylobacter* spp. from this country.

Considering this, the present study is aimed to describe the genomic traits associated with pathogenicity among representative* Campylobacter* spp. isolates recovered from faecal samples of patients with acute diarrhoea who were assisted in a hospital from the Chilean central area during the spring and summer 2017-2018.

## 2. Materials and Methods

All faecal samples of patients with acute diarrhoea assisted at the Hospital Naval Almirante Nef, Valparaíso, Chile, between October 2017 and April 2018 were collected. Acute diarrhoea was defined as more than 3 loose stools in 24 hrs and according to local recommendations, cultures were only processed in the following cases: patients in whom diarrhoea had persisted for more than 4-5 days, patients with bloody stools, immunocompromised patients, and patients who had recently travelled somewhere that possibly had an outbreak of enteric disease [25].

According to the routine protocol, samples were seeded onto SS, MacConkey, TCBS agar, and CASA (Biomerieux, France) for detection of enteropathogens such as* Salmonella* spp.,* Shigella* spp.,* Yersinia enterocolitica, Vibrio* spp., enteropathogenic* Escherichia coli*, and* Campylobacter *spp. The SS, MacConkey, and TCBS agar plates were incubated under aerobic conditions at 37°C for 24-48 h, and presumptive colonies of enteropathogens were identified with the VITEK 2 microbial identification system (Biomerieux, France) as described by the manufacturer.

Regarding the CASA plates, they were incubated under microaerobic atmosphere (~6% O2, 6% CO2, 3% H2, and 85% N2) using the Anaerocult® C generator system (Merck, USA) into an appropriate anaerobic jar at 42°C for 48-72h.

Typical red colonies on this medium were selected for phenotypical and molecular characterization at the Microbiology Laboratory of the Medical Technology School, Pontifical Catholic University of Valparaíso, Chile. For phenotypical characterization, the isolates were submitted to Gram staining, oxidase and hippuricase determination and motility observation.* Campylobacter* corresponded to Gram negative bacilli, curved, S or seagull shaped, positive for oxidase and motility. Those isolates that were also positive for hippuricase were identified as* C. jejuni*.

Once confirmed, the suspected colonies were streaked onto blood agar (BA, i.e., Trypticase Soya Agar supplemented with 5% sheep blood, Biomerieux, France), incubated under the same conditions and stored in 15% glycerol at -80°C.

For molecular confirmation, DNA was extracted from colonies using the InstaGene matrix (BioRad, USA) as indicated by manufacturer. The samples were submitted for genus-specific PCR for* Campylobacter* [26] using the commercial kit GoTaq Green Master Mix (Promega, USA) as previously described [27]. Afterwards, the isolates positive for genera* Campylobacter* were submitted for a second PCR for identification of species commonly associated with human infections:* C. jejuni, C. coli, C. ureolyticus, C. upsaliensis*, and* C. concisus* [27]. In addition, the identity of all isolates was confirmed by matrix-assisted laser desorption/ionization time-of-flight mass spectrometry (MALDI-TOF MS) using the Vitek MS equipment (Biomerieux, France) at the Clinical Laboratory of* Clínica Alemana*, Santiago, Chile.

The presence of potential virulence factors was also determined by PCR in all of the isolates identified as* Campylobacter* spp. (*iamA, pldA, cadF, cdtA, cdtB*, and* cdtC*) using the kit GoTaq Green Master Mix (Promega, USA), with the concentrations of primers and conditions described in the literature [14]. In order to control all the phenotypic and molecular tests, the type strains of* C. jejuni* subsp.* jejuni* DSM 4688^T^,* C. coli* DSM 4689^T^, and also* A. butzleri* LMG 10828^T^ were included in the analyses as controls. All PCR products were subjected to electrophoresis in 1% agarose gels (Sigma, USA) stained with SYBR® Safe DNA Gel Stain (Thermo Fisher, USA) and visualized in a UV transilluminator.

The susceptibility to antimicrobials was carried out with the Kirby-Bauer method using the MH-F medium (Müeller Hinton Agar supplemented with 5% defibrinated horse blood and *β*-NAD 20 mg/L, Liofilchem, Italy) for ampicillin, amoxicillin/clavulanic acid, gentamicin, ciprofloxacin, erythromycin, azithromycin, and tetracycline, using the strain* C. jejuni* subsp.* jejuni* DSM 4688^T^ (= ATCC 33560^T^) as control. All plates were incubated at 37°C for 48 h under microaerophilic conditions. In parallel, the minimum inhibitory concentration (MIC) was determined by the double dilution in agar method from 0.125 *μ*g/mL to 256 *μ*g/mL, for erythromycin, ciprofloxacin, tetracycline, and gentamicin (Sigma, USA) as previously described [28]. Cutoffs for interpretation of Kirby-Bauer and MIC results were obtained from the recommendations of the Committee for Antimicrobial Susceptibility Testing (AST) of the French Society for Microbiology [29].

For selection of representative isolates for whole genome sequencing (WGS), the* C. jejuni* isolates were genotyped by the* flaA*-RFLP method as previously described [28]. Briefly, the flagellin gene (*flaA*) was amplified and then digested with restriction enzyme* DdeI* (Promega, USA) as indicated by the manufacturer [28]. The digested PCR products were subjected to electrophoresis on 2% agarose gels (Sigma, USA) stained with SYBR® Safe DNA Gel Stain (Thermo Fisher, USA) and visualized in a UV transilluminator. The obtained profiles were analysed with the BioNumerics software version 7.0 (Applied Maths, Belgium) using the Dice coefficient and UPGMA algorithm. From this analysis, isolates that represented the most common* flaA*-RFLP genotypes were selected. For WGS, the selected isolates were grown in BA and incubated at 42°C for 48 hours under microaerophilic conditions, and the genomic DNA was extracted using the Wizard Genomic DNA purification kit (Promega, USA) as indicated by the manufacturer. Genomes were sequenced using Nextera XL and the NextSeq Illumina equipment at the Zooprophylactic Experimental Institute* dell'Abruzzo e del Molise “G. Caporale*,” Teramo, Italy. Basic bioinformatic analyses, quality control, genome assembly, and MLST determination were performed at the department of Food Hygiene and Environmental Health, Veterinary Faculty, University of Helsinki, Finland, using the INNUca pipeline (https://github.com/B-UMMI/INNUca) [30] and were annotated with Prokka [31].

The allele profiles for the wgMLST and cgMLST schema (Rossi et al., 2018; https://zenodo.org/record/1322564#.W3poK5MzYxc) were determined using the chewBBACA allele calling (https://www.ncbi.nlm.nih.gov/pubmed/29543149) and then were visualized using PHYLOViZ V2.0 software (https://online.phyloviz.net/index).

Searching for Antimicrobial Resistance (AMR) genes was performed by using “card” and “ResFinder” databases and Abricate software (https://github.com/tseemann/abricate). In addition, the* gyrA* was extracted using Artemis (https://www.sanger.ac.uk/science/tools/artemis) and single nucleotide mutations were directly searched by aligning the sequences with MEGA software [32]. Furthermore, BLAST 2.8.1+ was used to search for the virulence genes detected by PCR as well as other virulence genes described in the literature, i.e.,* wlaN, virB11, racR, pldA, iamB, dnaJ, ciaB, cgt, cfrA, cdtA, cdtB, cdtC, *and* cadF*. Moreover, the genetic determinants among* C. jejuni* lineages associated with human disease previously described by Buchanan et al. [24] were searched for by using a similar approach. Then, tblastn was used to find proteins in the genome of the 3 human isolates and in the reference genome of* C. jejuni*, strain NCTC 111168, GenBank AL11168.1. Finally, R was used with the pheatmap package (v1.0.11) to build a heatmap.

The obtained genomes were also compared to the reference genome of* C. jejuni* (strain NCTC 11168, GenBank AL11168.1), using the metabolic reconstruction comparison tool of SEED viewer version 2.0 at the RAST server (http://rast.theseed.org), which allows comparison of the functioning parts of two organisms.

In order to search for prophage sequences within genomes, the Phage Search Tool (PHAST) web server was used [33].

## 3. Results

A total of 350 samples were collected during the sampling time. The age of patients ranged between a few months and 91 years old (median 46 y, and mode 20 y); 172 (49%) corresponded to women and 178 (51%) to men.

**Figure 1:**
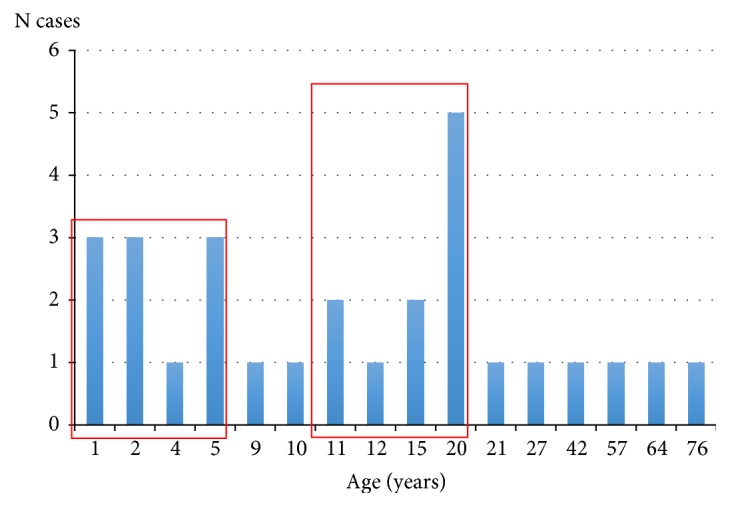
Distribution by the age of the 28 patients positive for* Campylobacter* spp. in this study. Red squares indicate group patients between 0-5 years old (n=10) and between 11-20 years old (n=10).

*Campylobacter* spp. was detected from 28 (8%) out of the 350 samples and* Salmonella* spp. was detected only from 10 (2.9%), while none of other bacterial enteropathogens were detected. The samples were positive for* Campylobacter* spp.; they were also negative for Rotavirus and Adenovirus as well as for known parasitic agents by using standard laboratory methods. The age of these patients ranged from 8 m to 76 y (median 11.5 y and mode 20 y), and most cases were concentrated in patients between 0 and 5 years old (n=10, 36%) and between 11 and 20 years old (n=10, 36%) (Figure 1). 13 patients (46%) corresponded to women and 15 (54%) to men (Figure 1). 26 (93%) of the* Campylobacter* isolates corresponded to* C. jejuni* and 2 (7%) to* C. coli*. One isolate of* C. jejuni* could not be recovered from storage for further analyses.

The presence of the* cdt *genes (A, B, and C) and* cadF* genes was detected by PCR in all the* C. jejuni* isolates recovered (n=25), while the* iam* gene was only detected in the* C. coli* isolates (Table S1).

Regarding the susceptibility to antibiotics, the interpretations of the results obtained by the Kirby-Bauer method were all confirmed by the minimum inhibitory concentration (MIC) determined by double dilution in agar for ciprofloxacin, erythromycin, tetracycline, and gentamicin (Tables 1 and S2). A high resistance to ciprofloxacin was observed among isolates (63%), followed by tetracycline (26%), while only one isolate (3.6%), belonging to* C. coli*, was resistant to erythromycin and none of them was resistant to gentamicin.

**Figure 2:**
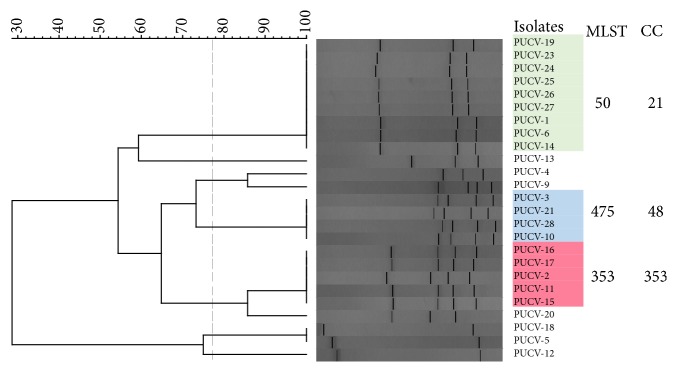
Dendrogram showing the clustering of the* flaA*-RFLP profiles of* C. jejuni *isolates from this study. The group represented by strains PUCV-1, PUCV-2, and PUCV-3 are shown in green, blue, and red, respectively.

**Figure 3:**
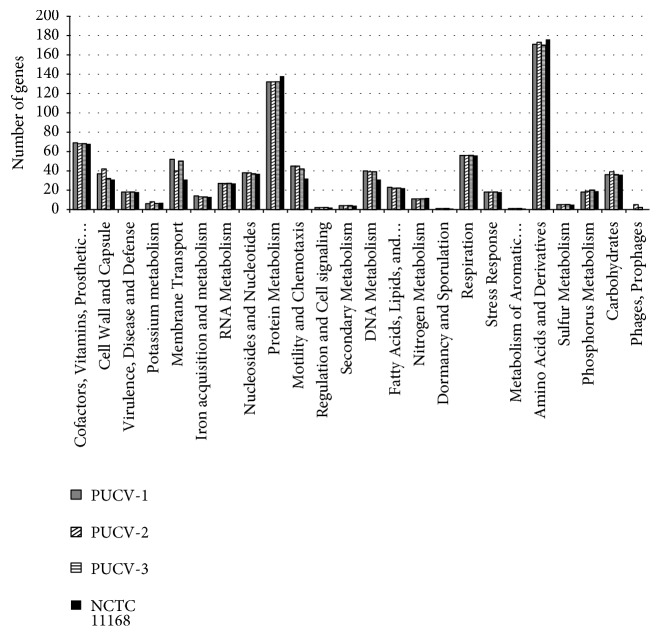
Functional classification of genes present in strains PUCV-1, PUCV-2, and PUCV-3 in comparison with strain NCTC 11168.

The 25* C. jejuni* isolates were genotyped by* flaA*-RFLP, and the obtained profiles were analysed with the BioNumerics software (Figure 2). On the basis of this method, most of the isolates (n=18, 72%, Figure 2) clustered in 3 groups. A representative isolate from each group was randomly selected for Whole Genome Sequencing. The characteristics of the sequences obtained from the selected strains, so-called PUCV-1, PUCV-2, and PUCV-3, are shown in Table 2. The three genomes were compared to the reference genome (NCTC 11168) on the basis of the functional classification of annotated genes using the RAST server. A similar distribution of genes was observed among all genomes compared, and most of genes were grouped in the categories of protein metabolism and amino acids and derivatives (Figure 3). Interestingly, using the metabolic reconstruction comparison tool of SEED viewer version 2.0 at the RAST server, PUCV-1 and PUCV-3 but not the reference genome (NCTC 11168) or PUCV-2 have the complete Type IV Secretion System (T4SS) encoded within* virB* type plasmids (Table S4). Moreover, only the strain PUCV-2 possessed a complete Type VI secretion system (T6SS, Table S4).

The strains PUCV-1, PUCV-2, and PUCV-3, as well as the reference strain, possessed prophage sequences. However, only PUCV-3 has a complete phage of 19.3 kb, which showed similarity with phages present in several bacterial species (Table S5). Most bacterial proteins encoded in the detected phages corresponded to hypothetical proteins of* C. jejuni *(Table S5).

**Figure 4:**
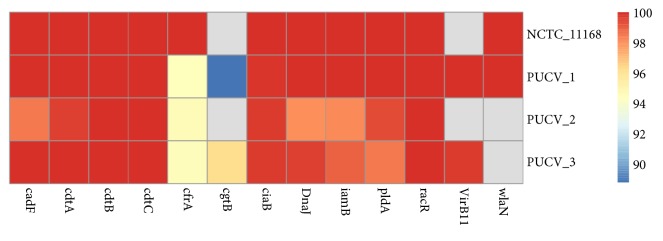
Heatmap obtained using the pheatmap package (v1.0.11), showing the presence of virulence associated genes detected in the Chilean strains and the reference genome of* C. jejuni* (strain NCTC 11168, GenBank AL11168.1, 70% coverage and 90% identity). Scale at the right indicates % identity of detected genes, while gray indicates no detection.

**Figure 5:**
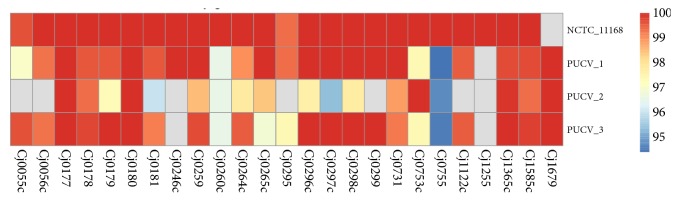
Heatmap obtained using the pheatmap package (v1.0.11), showing the presence of the genetic determinants associated with human disease described by Buchanan et al.[24] in the Chilean strains and the reference genome of* C. jejuni* (strain NCTC 11168, GenBank AL11168.1). Scale at the right indicates % identity of detected genes, while gray indicates no detection.

The strain PUVC-1 belonged to ST-50 (CC-21), strain PUCV-2 to ST-353 (CC-353), and PUCV-3 to ST-475 (CC-48). The cgMLST and wgMLST obtained for these representative strains were obtained with the chewBBACA allele calling and clustered together with the 6526 genomes from strains* C. jejuni *contained in the INNUca V3.1 database (Rossi et al., 2018; https://zenodo.org/record/1322564#.W3poK5MzYxc) using the PHYLOViZ V2.0 software. The three strains clustered mainly with clinical isolates from humans. However, PUCV-1 clustered with genome SRR2727700 which was recovered in the USA from ruminant (*Bos taurus*), while PUCV-2 clustered with SRR1811817, recovered in the UK from poultry (*Gallus gallus*) and PUCV-3 clustered with IN_Cj_FI_273, recovered in the USA from poultry (*G. gallus*) (Table S3). In the search results for virulence genes, 14 virulence genes were detected in strain PUCV-1, 13 in PUCV-3, and only 10 were detected in PUCV-2 (Figure 4). Furthermore, the genetic determinants among* C. jejuni* lineages associated with human disease previously described by Buchanan et al. [24] were searched for in the 3 genomes (Figure 5). In strain PUCV-1, 24 out of the 25 genes were detected, while 23 were detected in PUCV-3, and only 18 were detected in PUCV-2. Regarding the genes associated with AMR, the results obtained with ResFinder and CARD databases were similar (Table 3). Seven genes associated with AMR were detected in PUCV-1, while in PUCV-2 and PUCV-3, five genes were detected.

Moreover, in PUCV-1, the single nucleotide mutation Thr86Ile at the* gyrA* gene was also detected, which has been previously associated with resistance to fluoroquinolones [20].

## 4. Discussion

*Campylobacter* spp. (8%), especially* C. jejuni*, are the bacterial pathogens most commonly isolated from the studied samples followed by* Salmonella* spp. (2.9%). This difference in the prevalence of both genera was not expected because in a previous study, almost the same prevalence of* Campylobacter *(6.1%) and* Salmonella* (6.5%) was detected from stool samples [11]. However, this is the first report from the Valparaíso region, Chile, and a different epidemiological distribution of enteropathogens between this and other geographical regions cannot be ruled out. No difference was observed in the distribution of isolates between men and women. Regarding the distribution by the age of patients, most of them were grouped into two ranges, i.e., between 0 and 5 years old and between 11 and 20 years old, and the median and mode were 11.5 and 20, respectively. These results are not consistent with results obtained in developing countries, where* Campylobacter *diarrhoea is seen in children under 5 years old, but rarely in adults [34].

In the present study, no clinical information has been gathered from the Medical History of the patients, as the authorization for this action was not requested to the local ethics committee. Despite this, the local recommendations [25] indicate that stool culture is only indicated in case of acute diarrhoea, and the 350 patients included in this study comply with this recommendation. Moreover, in 14 cases, Faecal Leukocyte Test (FLT) was determined, and 9 of them (64.3%) were positive, including PUCV-1, PUCV-2, and PUCV-3 (Table S1). Although the utility of FLT has been discussed due to its inability to estimate stool culture results and response to antimicrobials, not necessarily their inability to detect inflammation, which may have many causes, the association between the presence of faecal leukocytes and exudative enteropathy such as ulcerative colitis, Crohn's disease, amoebic colitis, eosinophilic colitis, and even colonic carcinoma has been previously demonstrated [35, 36].

As determined by PCR, all the* C. jejuni* isolates presented the Cytotoxic Distending Toxin (*cdt* gene subunits A, B, and C), associated with cell cycle arrest as well as* pldA, *and* cadF* genes which are associated with cell adhesion and invasion [13–15]. In a previous study among 73 Chilean isolates of* Campylobacter *spp., the most prevalent genes were* cadF* (93%),* cdtC*, and* cdtB* (85%) [19]. Although the role of these genes in pathogenesis is not completely elucidated, their higher prevalence in isolates from human origin compared with animal origin or food indicates that the degree of virulence between these sources differs [19]. Regarding CDT, it has been suggested that this genotoxin, which induces DNA double-strand breaks, could lead to an increased risk of cancer, especially in the gastrointestinal tract. In this sense, Brauner et al. [37] observed no excess risks of malignancies following an infection by* C. jejuni* among all individuals in Stockholm County who tested positive with* C. jejuni* between 1989 and 2006. Furthermore, they also observed a decreased risk of respiratory cancers among the cohort. However, more recently He et al. [38] demonstrated that* C. jejuni* promotes colorectal tumorigenesis through the action of CDT. These authors observed that GF *Apc*^Min/+^mice colonized with the human clinical isolate* C. jejuni *81–176 developed significantly more and larger tumours when compared with uninfected mice, which could be diminished by rapamycin. Moreover,* C. jejuni *with a mutated* cdtB *subunit (mut*cdtB*) showed an attenuated* C. jejuni*-induced tumorigenesis in vivo and a decreased DNA damage response in cells and enteroids [38]. Furthermore,* C. jejuni *infection induced expression of hundreds of colonic genes, with 22 genes dependent on the presence of c*dtB *[38].

A high resistance to ciprofloxacin was observed among isolates (63%), followed by tetracycline (25.9%), and only one isolate of* C. coli* was resistant to erythromycin (3.7%). Previous studies have shown an increasing resistance to ciprofloxacin among Chilean isolates. No resistance was observed among* C. jejuni *clinical strains isolated between 1996 and 1997 by Fernández et al. [39], while resistance to this antibiotic as high as 48% was reported later on by Collado et al. [21]. It has been stated that food-producing animals and the food chain are the main source for transmission of fluoroquinolone-resistant strains in Latin American countries [12]. However, more studies to determine the sources of this increasing resistance to fluoroquinolones are warranted. In relation to erythromycin and gentamicin, on the basis of our results, they both remain useful for treatment of campylobacteriosis in Chile.

As indicated, a good correlation was observed between the results obtained by the Kirby-Bauer method and the minimum inhibitory concentration (MIC) determined by double dilution in agar for ciprofloxacin, erythromycin, tetracycline, and gentamicin (Tables 1 and S2). Considering the current need for AMR surveillance in* Campylobacter* spp., our results indicate that the Kirby-Bauer method, which is less expensive and easy to perform than MIC methods, could be used in most of laboratories to implement the surveillance on the susceptibility of these 4 antibiotics in these bacteria.

WGS analyses of bacterial pathogens have been useful for the current understanding of the pathogenic and resistance determinants of* Campylobacter* spp. However, most of studies have been conducted by comparing genomes from developed countries and, to our knowledge, isolates from Chilean or other Latin American patients have not been included in those analyses. Therefore, we selected representative genomes from this set of isolates in order to obtain relevant information such as virulence determinants, AMR-associated genes, and possible sources of the isolates that circulate in this country. For selection of the isolates for WGS analyses, the Restriction Fragment Length Polymorphism of* flaA* gene (*flaA*-RFLP) was used. This genotyping method was selected because it is a cost-efficient typing technique widely used, which is based on the amplification of the flagellin gene (*flaA*) of* C. jejuni* and its subsequent digestion with a restriction enzyme, especially the* DdeI* enzyme which has been able to differentiate about 90% of the strains of this species [28]. Based on the good results obtained with this technique, Meinersmann et al. [40] studied the usefulness of the analysis by sequencing the complete sequence of the* flaA* gene, as well as the hypervariable zone located between positions 450 and 600bp, known as* flaA*-SVR. The latter showed a good ability to discriminate isolates and in a more recent study, the results obtained with this technique were comparable to those obtained by MLST [41]. The three isolates selected for WGS on this basis (PUCV-1, PUCV-2, and PUCV-3) represented the* flaA*-RFLP groups that included more than 70% of the isolates (Figure 2). In the functional comparison of their genomes by using the RAST server, a similar distribution of genes among the three strains was observed (Figure 3). Among these genomes, two functional categories were the most represented, i.e., the protein metabolism and amino acids and derivatives, while the sugar fermentation category was less represented (Figure 3). This was expected because in genus* Campylobacter*, amino acids are essential for energy production and also as a carbon source.

Those strains were assigned to their respective ST and they belonged to ST-50 (CC-21, PUCV-1), ST-475 (CC-48, PUCV-3), and ST-353 (CC-353, PUCV-2), respectively. In a previous study from Santiago, Chile, four predominant clonal complexes (ST-21, ST-48, ST-257, and ST-353) were the dominant types among the human* C. jejuni *strains in various geographic Chilean regions [21]. In that study, the authors observed an association between the predominant CC and AMR (from 66.7% to 80% of strains resistant to ciprofloxacin), suggesting that the dissemination and expansion of resistant clonal lineages are responsible for cases of human campylobacteriosis in Chile [21]. However, despite the fact that in the present study a different genotyping method was used to cluster the genotypes (*flaA*-RFLP), a different distribution of resistance was observed among isolates related to CC-21 (100%), CC-48 (20%), and CC-353 (25%), suggesting that only CC-21 is associated with this trait. As expected, the strain PUCV-1 (CC-21) also showed the presence of several genes associated with AMR as well as the single nucleotide mutation in* gyrA* gene, which leads to fluoroquinolone resistance.

It should be noted that the genomes of the 3 Chilean strains have the* cme* genes (subunits A, B, C, and the regulatory gene R), which encoded an efflux pump associated with resistance to several antibiotics including those tested in this study. However, their presence did not affect the AMR in our isolates probably due to the fact that their effect is synergistic with other factors and this was not assessed in this study [42]. Regarding the* lnuC* gene, it encodes a transposon-mediated nucleotidyl transferase that inactivates lincomycin and clindamycin in the presence of ATP and MgCl_2_, [43]. Those antibiotics were not tested; therefore, the role of this gene among our strains needs to be further studied. Regarding the aminoglycoside-9 nucleosidyl transferase detected in PUCV-1, the presence of this gene did not affect the phenotypic resistance to aminoglycosides (gentamicin CIM 0.125, Table S2). However, whether the resistance to gentamicin or other aminoglycosides could be induced in vivo or in vitro remain to be studied. Within the functional category of resistance to antibiotics and toxic compounds, the three sequenced strains showed the presence of genes associated with copper homeostasis and tolerance, as well as cobalt-zinc-cadmium resistance (data not shown). Considering that currently there is an intense interest in the use of copper as a hygienic material in hospitals and related settings due to its capacity of contact-mediated killing of pathogenic bacteria [44], the role of these genes in copper resistance needs further attention.

The plasmidic* cfr(C) *gene, recently described by Tang et al. [45], which confers multidrug resistance in* Campylobacter *to five chemically unrelated antimicrobial classes including phenicols, lincosamides, oxazolidinones, pleuromutilins, and streptogramin A (known as the PhLOPS_A_ phenotype) was searched for and it was not detected among the sequenced strains.

In order to assess in silico the presence of virulence determinants, a set of 38 genes were searched for in the 3 Chilean strains in comparison with the reference genome of* C. jejuni*. 25 out of these genes were previously identified as robust putative diagnostic markers for clinical related* C. jejuni* strains by Buchanan et al. [24] among isolates mainly from Canada and the United Kingdom. Although many of these 25 genes still have unknown function, some of them had previously shown a role in important processes such as iron acquisition and vitamin B5 biosynthesis. In our study, the strain PUCV-1 (CC-21) also showed the higher distribution of those genes (24/25), while PUCV-2 showed the lower distribution (18/25). The 7 genes, which were not detected in the latter strain, corresponded to* Cj1255* (putative isomerase),* Cj1122c *(putative integral membrane),* Cj0299* (putative periplasmic beta-lactamase),* Cj0295* (putative acetyltransferase), and* Cj0246c* (Putative MCP-domain signal transduction protein), while* Cj0055c* and* Cj0056c* were hypothetical proteins [24]. It is possible that the low distribution observed in PUCV-2 is due to the different geographical isolation as the validation of putative markers was performed in a dataset mainly from UK (90%) and Canada (8%), but also it could be due to the restrictive threshold used (99% identity, 90% coverage). However, based on these results, there are still several of the genes proposed by Buchanan et al. [24] that could be used as diagnostic markers for clinical related* C. jejuni* strains from this country.

Another important question that motivates the present study is where the* Campylobacter* strains that affect Chilean patients are coming from. In order to answer this question, the possible origin of the strains PUCV-1, PUCV-2, and PUCV-3 was assessed by searching the cgMLST and wgMLST with the chewBBACA allele calling and clustering with the 6526 genomes from strains* C. jejuni *contained in the INNUca V3.1 database. Based on this analysis, PUCV-1 (CC-21) was linked with* Bos taurus* (ruminant), while PUCV-2 and PUCV-3 were linked with* Gallus gallus* (poultry). Although the low number of genomes analysed could not be representative, these results suggest that food of animal origin could be the main source for campylobacteriosis in the studied isolates. This is relevant considering that in developing countries, campylobacteriosis has been associated with the exposure to the environment, including contaminated drinking water, while contaminated food, including poultry, are typically linked with these infections in developed countries [34]. In other words, our results suggest the epidemiology of campylobacteriosis in Chile is similar to that observed in developed countries, which is not surprising considering that the World Bank classifies Chile as a High-Income Economy. In this line, similar studies should be conducted in other Latin American countries in order to clarify the epidemiology and transmission dynamics in this part of the world.

On the other hand, the combination of the high ciprofloxacin resistance observed among Chilean isolates and their possible association with animal origin provides new evidence supporting the statement that food-producing animals and the food chain is the main source for transmission of resistant strains in Latin American countries [12].

It needs to be highlighted that the strain which represents the most prevalent* flaA*-RFLP genotype in this study is PUCV-1 and it belongs to CC-21 that is known as a generalist lineage [46]. This means that this CC is found from a wide variety of sources and reservoirs worldwide and often belongs to the top three most common lineages in large molecular epidemiological* C. jejuni* studies [46]. Therefore, this capacity for host adaptation could explain the higher AMR observed among the isolates belonging to this genotype as well as the higher number of virulence associated genes observed in PUCV-1 in comparison with PUCV-2 and PUCV-3.

A complete Type IV Secretion System (T4SS) encoded by plasmids is present in the strains PUCV-1 and PUCV-3. Previously, Bacon et al. [47] detected a gene of this plasmid (*virB11*) in about 10% of fresh clinical isolates of* C. jejuni *and observed that the mutation of two genes of this structure resulted in a 6-fold reduction in adherence and 11-fold reduction in invasion compared to the wild type. More recently, this structure has been detected only in 8 to 31% WGS analysed by Weis et al. [22]. Therefore, it is noteworthy that two out of three Chilean strains sequenced in this study possessed the genes encoding this structure and whether this is a tendency among Chilean strains needs to be demonstrated. Following the classification of Marasini et al. [48], the sequence of PUCV-1 strain is compatible with a Type-1 (pTet) plasmid as it includes all the 23 genes of the core genome described, including the genes for the T4SS,* virB2, virB4, virB5, virB6, virB7, virB8, virB9, virB10*, and* virB11* and the* tetO*, encoding a tetracycline resistance protein [48]. On the other hand, on the basis of the classification of Marasini et al. [48], the strain PUCV-3 possessed a smaller plasmid which could be classified as Type-3 (pVir) plasmid, whose core genome includes those genes encoding a single-stranded DNA binding protein,* topA, virB11, virB10, virB9, virB8, virB4, traQ, repE*, and* parA*. The T4SS genes are responsible for conjugation and are often encoded on self-transmissible plasmids together with genes that provide selective advantage for the cell such as antibiotic resistance, virulence traits, or other metabolic functions and sometimes deliver oncogenic nucleoprotein complexes into host cells [49]. Another type of T4SS mediates DNA uptake and release from the extracellular milieu, while a third type is used to transfer virulence proteins [49]. The conjugative function seems to be conserved in* Campylobacter *spp., which also indicates that it is possible that it might transfer conjugative genes among nonrelated microbes, and they frequently contain the* tetO* gene or other resistance genes [48]. However, the presence of T4SS in the genome of the clinical strains sequenced warrants the study of other possible functions of this structure in* Campylobacter* spp. Moreover, Marasini et al. [48] observed that plasmids found in* Campylobacter *species likely have a convoluted evolutionary history with intraspecies dissemination and indicated that additional sampling will be needed to more fully understand their evolution and transmission. In this regard, our study provides new sequences that could be included in those analyses.

In the case of PUCV-2, all the genes previously listed by Lertpiriyapong et al. [50] of a Type VI Secretion System (T6SS) were detected. T6SS consists of a membrane-associated assembly platform and a needle structure that shows similarity to elements of tailed bacteriophages that transports effector molecules into neighbouring bacteria or eukaryotic cells [51]. The T6SS of proteobacteria has been shown to promote pathogenicity, competitive advantage over competing microorganisms, and adaptation to environmental perturbation [50]. However, in* Campylobacter,* the role in pathogenicity of T6SS is controversial. On the one hand, Agnetti et al. [52] detected the T6SS in 16.8% cases of* C. jejuni* infections and observed no evidence of its association with a more severe clinical course, but these isolates were more commonly found in immunocompromised patients which merits further investigation. On the other hand, Lertpiriyapong et al. [50] by means of* in silico*,* in vitro*, and* in vivo* analyses demonstrated that the T6SS in* C. jejuni* is functional and it exerts pleiotropic effects on two crucial processes: survival in a bile salt, deoxycholic acid (DCA), and host cell adherence and invasion. Furthermore, Bleumink-Pluym et al. [51] using a proteomic and genetic analysis observed a contact-dependent cytotoxicity towards red blood cells, but not macrophages in capsule-deficient bacteria that represented a novel evolutionary pathway of T6SS in bacteria and expands the repertoire of virulence properties associated with T6SS.

Finally, the strains PUCV-1, PUCV-2, and PUCV-3, as well as the reference strain, possessed prophage sequences, but most of them were incomplete. Most of the bacterial proteins encoded by those phages corresponded to hypothetical proteins of* C. jejuni *(Table S4). It has been stated that integration of prophages into bacterial chromosome can modify the lifestyle, fitness, virulence, and evolution of their bacterial host in numerous ways, and whole bacterial genomic sequences provide a great opportunity to seek novel prophage sequences [53].

In the present study, a set of* Campylobacter* spp. isolates was characterized, and three representative strains were selected for WGS on the basis of their* flaA*-RFLP genotype. These strains which belonged to CC-21, CC-48, and CC-353 are the first genomes of Chilean* C. jejuni* isolates available in public databases and they provide relevant information about the genetic determinants of virulence, AMR, and possible origin of the* C. jejuni* associated with human infection in this country.

## Figures and Tables

**Table 1:** Percent distribution of Minimal Inhibitory Concentration (MIC) among Chilean isolates. MIC values under or at the cutoff for each antibiotic are shown in bold.

Antibiotic	% of isolates with each Minimal Inhibitory Concentration (*µ*g/ml)										
	0.0625	0.125	0.25	0.5	1	2	4	8	16	32	64
Ciprofloxacin	**25.9**	**11.1**								37.0	25.9
Erythromycin					**29.6**	**63.0**		**3.7**	3.7		
Tetracycline		**3.7**	**22.2**	**22.2**	**18.5**	**7.4**	11.1	3.7	11.1		
Gentamicin		**59.3**	**7.4**	**7.4**	**14.8**	**11.1**					

**Table 2:** Sequencing metrics and genomic data obtained for *Campylobacter jejuni* strains PUCV-1, PUCV-2, and PUCV-3.

Feature	PUCV-1	PUCV-2	PUCV-3
Source	Human faeces	Human faeces	Human faeces
BioProject no.	PRJNA531695	PRJNA531695	PRJNA531695
SRA accession no.	SSLZ00000000	SSMA00000000	SSMB00000000
Sequencing metrics			
Raw coverage (x)	209	226.5	167.2
Assembly coverage	164.99	169.82	122.57
Genomic data			
Size (bp)	1671271	1771578	1721012
No. of contigs	43	52	34
G+C content (%)	30.4	30.3	30.4
N50	155061	153515	116260
L50	3	4	4
No. of CDS	1769	1947	1761
No. of subsystems	204	210	206
No. of RNAs	42	42	42

**Table 3:** Percent of identity of AMR-associated genes and single nucleotide mutation in *gyrA* gene (*gyrA* snm) detected among Chilean strains.

Strain	*oxa61*	*cmeA*	*cmeB*	*cmeC*	*cmeR*	*lnu(C)*	*tetO*	*gyrA* snm
PUCV-1	100	100	100	100	100	100	100	Thr86Ile
PUCV-2	-----	100	99.9	100	100	100	-----	-----
PUCV-3	100	100	100	100	100	-----	-----	-----

## Data Availability

The data used to support the findings of this study are available from the corresponding author upon request.

## Supplementary Materials

Supplementary Materials Table S1. Distribution of putative virulence genes among Chilean isolates as detected by PCR. Table S2. Distribution of susceptibility results among Chilean isolates by Double Dilution in Agar (CIM) and Kirby Bauer (KB) methods and Faecal Leukocytes Test (FLT) when determined (ND=not determined). Isolates have been grouped by* flaA-*RFLP groups. Amox-CA: Amoxicillin Clavulanic Acid. Table S3. Multilocus Sequence Typing genotype (MLST), Clonal Complex (CC) and origin of the isolates that clustered with Chilean strains in the PHYLOVIZ 2.0 software on the basis of their wgMLST. NI: not indicated. Origins different than clinical are in bold. Table S4. Comparison between Chilean strains and the reference genome of* C. jejuni *(strain NCTC 11168, GenBank AL111168.1), using the metabolic reconstruction comparison tool of SEED viewer version 2.0 at the RAST server. Table S5. Summary of prophage sequences found within genomes of* C. Jejuni* strains studied using the PHAST software.
