# Supplementary material for: Genomic Analysis of Chilean Strains of Campylobacter jejuni from Human Faeces
Source: Biomed Res Int. 2019 Jul 8;2019:1902732. doi: 10.1155/2019/1902732 (PMC6644508; doi:10.1155/2019/1902732)
Supplement: Supplementary Materials — Table S1. Distribution of putative virulence genes among Chilean isolates as detected by PCR. Table S2. Distribution of susceptibility results among Chilean isolates by Double Dilution in Agar (CIM) and Kirby Bauer (KB) methods and Faecal Leukocytes Test (FLT) when determined (ND=not determined). Isolates have been grouped by flaA-RFLP groups. Amox-CA: Amoxicillin Clavulanic Acid. Table S3. Multilocus Sequence Typing genotype (MLST), Clonal Complex (CC) and origin of the isolates that clustered with Chilean strains in the PHYLOVIZ 2.0 software on the basis of their wgMLST. NI: not indicated. Origins different than clinical are in bold. Table S4. Comparison between Chilean strains and the reference genome of C. jejuni (strain NCTC 11168, GenBank AL111168.1), using the metabolic reconstruction comparison tool of SEED viewer version 2.0 at the RAST server. Table S5. Summary of prophage sequences found within genomes of C. Jejuni strains studied using the PHAST software. [file 1902732.f1.pdf]

Table S1. Distribution of putative virulence genes among Chilean isolates as detected by PCR

		cdtA	*cdtB*	*cdtC*	*cadF*	*pldA*	*iam*
**PUCV-1**	*C. jejuni*	+	+	+	+	+	-
**PUCV-2**	*C. jejuni*	+	+	+	+	+	-
**PUCV-3**	*C. jejuni*	+	+	+	+	+	-
**PUCV-4**	*C. jejuni*	+	+	+	+	+	-
**PUCV-5**	*C. jejuni*	+	+	+	+	+	-
**PUCV-6**	*C. jejuni*	+	+	+	+	+	-
**PUCV-7**	*C. coli*	-	-	-	+	-	+
**PUCV-8**	*C. coli*	-	-	-	+	-	+
**PUCV-9**	*C. jejuni*	+	+	+	+	-	-
**PUCV-10**	*C. jejuni*	+	+	+	+	+	-
**PUCV-11**	*C. jejuni*	+	+	+	+	+	-
**PUCV-12**	*C. jejuni*	+	+	+	+	-	-
**PUCV-13**	*C. jejuni*	+	+	+	+	+	-
**PUCV-14**	*C. jejuni*	+	+	+	+	-	-
**PUCV-15**	*C. jejuni*	+	+	+	+	+	-
**PUCV-16**	*C. jejuni*	+	+	+	+	+	-
**PUCV-17**	*C. jejuni*	+	+	+	+	+	-
**PUCV-18**	*C. jejuni*	+	+	+	+	+	-
**PUCV-19**	*C. jejuni*	+	+	+	+	+	-
**PUCV-20**	*C. jejuni*	+	+	+	+	+	-
**PUCV-21**	*C. jejuni*	+	+	+	+	+	-
**PUCV-23**	*C. jejuni*	+	+	+	+	+	-
**PUCV-24**	*C. jejuni*	+	+	+	+	+	-
**PUCV-25**	*C. jejuni*	+	+	+	+	+	-
**PUCV-26**	*C. jejuni*	+	+	+	+	+	-
**PUCV-27**	*C. jejuni*	+	+	+	+	+	-
**PUCV-28**	*C. jejuni*	+	+	+	+	+	-

Table S2. Distribution of susceptibility results among Chilean isolates by Double Dilution in Agar (CIM) and Kirby Bauer (B) methods and Fecal Leukocites Test (FLT) when determined (ND=Not determined). Isolates have been grouped by flaA-RFLP groups. Amox-CA, Amoxicilin Clavulanic Acid.

		Erythromycin		Ciprofloxacin		Tetracyclin		Gentamicin		Amox-CA	Ampicilin	FLT
	**Species**	**CIM**	**KB**	**CIM**	**KB**	**CIM**	**KB**	**CIM**	**KB**	**KB**	**KB**	
**PUCV-1**	*C. Jejuni*	2,00	S	64,00	R	16,00	R	0.125	S	S	R	Positive
**PUCV-6**	*C. Jejuni*	2,00	S	32,00	R	0,50	S	0,13	S	S	R	ND
**PUCV-14**	*C. Jejuni*	2,00	S	32,00	R	0,25	S	0,13	S	S	R	ND
**PUCV-19**	*C. Jejuni*	2,00	S	32,00	R	1,00	S	2,00	S	R	R	Positive
**PUCV-23**	*C. Jejuni*	1,00	S	32,00	R	2,00	S	1,00	S	R	R	ND
**PUCV-24**	*C. Jejuni*	2,00	S	32,00	R	4,00	R	2,00	S	R	R	Positive
**PUCV-25**	*C. Jejuni*	2,00	S	32,00	R	2,00	S	0,13	S	I	R	ND
**PUCV-26**	*C. Jejuni*	2,00	S	32,00	R	4,00	R	0,13	S	I	R	ND
**PUCV-27**	*C. Jejuni*	2,00	S	64,00	R	1,00	S	0,25	S	I	R	ND
**PUCV-2**	*C. Jejuni*	1,00	S	0,06	S	4,00	R	1,00	S	S	S	Positive
**PUCV-11**	*C. Jejuni*	1,00	S	0,06	S	0,25	S	0,13	S	S	S	Negative
**PUCV-15**	*C. Jejuni*	2,00	S	0,06	S	0,13	S	0,13	S	S	S	ND
**PUCV-16**	*C. Jejuni*	2,00	S	0,06	S	0,25	S	0,13	S	S	S	ND
**PUCV-17**	*C. Jejuni*	2,00	S	0,06	R	1,00	S	1,00	S	S	R	ND
**PUCV-3**	*C. Jejuni*	1,00	S	0,13	S	0,50	S	0,13	S	S	S	Positive
**PUCV-10**	*C. Jejuni*	2,00	S	64,00	R	0,25	S	0,50	S	S	R	ND
**PUCV-21**	*C. Jejuni*	2,00	S	0,13	S	0,50	S	1,00	S	R	R	ND
**PUCV-28**	*C. Jejuni*	1,00	S	0,06	S	1,00	S	0,25	S	S	I	Positive
**PUCV-4**	*C. Jejuni*	2,00	S	64,00	R	8,00	R	0,13	S	S	R	Negative
**PUCV-5**	*C. Jejuni*	2,00	S	64,00	R	16,00	R	0,13	S	S	R	Positive
**PUCV-7**	*C. Coli*	8,00	S	32,00	R	0,50	S	0,13	S	S	R	ND
**PUCV-8**	*C. Coli*	16,00	R	32,00	R	0,50	S	0,13	S	S	R	Positive
**PUCV-9**	*C. Jejuni*	0,50	S	0,06	S	0,50	S	0,13	S	S	S	Negative
**PUCV-12**	*C. Jejuni*	2,00	S	32,00	R	16,00	R	0,13	S	S	R	Negative
**PUCV-13**	*C. Jejuni*	1,00	S	0,13	S	0,25	S	0,13	S	S	R	ND
**PUCV-18**	*C. Jejuni*	2,00	S	64,00	R	1,00	S	2,00	S	S	R	Negative
**PUCV-20**	*C. Jejuni*	1,00	S	64,00	R	0,25	S	0,50	S	S	R	Positive

Table S3. Multilocus Sequence Typing genotype (MLST), Clonal Complex (CC) and origin of the isolates that clustered with Chilean strains in the PHYLOVIZ 2.0 software on the basis of their wgMLST. NI, Not indicated; Origins different than clinical are in bold.

Strain	Country	MLST	CC	Origin	Host species
**PUCV-1**	Chile	50	21	Clinical	*Homo sapiens*, this study
**SRR3215187**	USA	50	21	Clinical	*Homo sapiens*
**SRR1993533**	USA	50	21	Clinical	*Homo sapiens*
**SRR1999657**	USA	50	21	Clinical	*Homo sapiens*
**SRR3215189**	USA	50	21	Clinical	*Homo sapiens*
**ERR189826**	UK	536	21	Clinical	*Homo sapiens*
**SRR2727700**	**USA**	**50**	**21**	**Ruminant**	***Bos taurus***
**ERR736711**	NI	50	21	NI	NI
**ERR348972**	UK	50	21	Clinical	*Homo sapiens*
**PUCV-3**	Chile	475	48	Clinical	*Homo sapiens*, this study
**SRR1816214**	NI	475	48	NI	NI
**SRR1975114**	USA	475	48	Clinical	*Homo sapiens*
**SRR1772864**	USA	475	48	Clinical	*Homo sapiens*
**ERR189832**	NI	475	48	NI	NI
**SRR1816209**	NI	475	48	NI	NI
**IN_CJ_FI_273.fasta**	**Finland**	**475**	**48**	**Poultry**	***Gallus gallus***
**ERR757413**	NI	918	48	NI	NI
**PUCV-2**	Chile	353	353	Clinical	*Homo sapiens*, this study
**ERR330473**	UK	353	353	Clinical	*Homo sapiens*
**SRR2157043**	USA	353	353	Clinical	*Homo sapiens*
**ERR494578**	UK	353	353	Clinical	*Homo sapiens*
**SRR2938812**	USA	353	353	Clinical	*Homo sapiens*
**SRR3380856**	USA	NI	NI	NI	NI
**ERR108370**	UK	353	353	Clinical	*Homo sapiens*
**SRR3380866**	USA	1953	353	Clinical	*Homo sapiens*
**SRR1811817**	**USA**	**353**	**353**	**Poultry**	***Gallus gallus***

Table S4. Comparison between Chilean strains and the reference genome of *C. jejuni* (strain NCTC 11168, GenBank AL111168.1), using the metabolic reconstruction comparison tool of SEED viewer version 2.0 at the RAST server.

Category	Subcategory	Subsystem	Role
**Table S4-A**	**PUCV-1 vs reference genome of *C. Jejuni* (NCTC 111168)**		
Cell Wall and Capsule	Capsular and extracellular polysacchrides	Capsular heptose biosynthesis	Capsular polysaccharide biosynthesis heptosyltransferase HddD
Cell Wall and Capsule	Capsular and extracellular polysacchrides	Capsular heptose biosynthesis	GDP-mannose 4,6-dehydratase (EC 4.2.1.47)
Cofactors, Vitamins, Prosthetic Groups, Pigments	Biotin	Biotin biosynthesis	Long-chain-fatty-acid--CoA ligase (EC 6.2.1.3)
DNA Metabolism	DNA replication	DNA topoisomerases, Type I, ATP-independent	DNA topoisomerase III, TraE-type (EC 5.99.1.2)
Membrane Transport	Protein and nucleoprotein secretion system, Type IV	Conjugative transfer	IncQ plasmid conjugative transfer DNA nicking endonuclease TraR (pTi VirD2 homolog)
Membrane Transport	Protein and nucleoprotein secretion system, Type IV	Conjugative transfer	IncQ plasmid conjugative transfer protein TraG
Membrane Transport	Protein and nucleoprotein secretion system, Type IV	Conjugative transfer	IncQ plasmid conjugative transfer protein TraQ (RP4 TrbM homolog)
Membrane Transport	Protein and nucleoprotein secretion system, Type IV	Vir-like type 4 secretion system	ATPase required for both assembly of type IV secretion complex and secretion of T-DNA complex, VirB11
Membrane Transport	Protein and nucleoprotein secretion system, Type IV	Vir-like type 4 secretion system	ATPase required for both assembly of type IV secretion complex and secretion of T-DNA complex, VirB4
Membrane Transport	Protein and nucleoprotein secretion system, Type IV	Vir-like type 4 secretion system	Coupling protein VirD4, ATPase required for T-DNA transfer
Membrane Transport	Protein and nucleoprotein secretion system, Type IV	Vir-like type 4 secretion system	Inner membrane protein forms channel for type IV secretion of T-DNA complex, VirB3

Membrane Transport	Protein and nucleoprotein secretion system, Type IV	Vir-like type 4 secretion system	Inner membrane protein forms channel for type IV secretion of T-DNA complex, VirB8
Membrane Transport	Protein and nucleoprotein secretion system, Type IV	Vir-like type 4 secretion system	Inner membrane protein of type IV secretion of T-DNA complex, TonB-like, VirB10
Membrane Transport	Protein and nucleoprotein secretion system, Type IV	Vir-like type 4 secretion system	Inner membrane protein of type IV secretion of T-DNA complex, VirB6
Membrane Transport	Protein and nucleoprotein secretion system, Type IV	Vir-like type 4 secretion system	Lipoprotein of type IV secretion complex that spans outer membrane and periplasm, VirB7
Membrane Transport	Protein and nucleoprotein secretion system, Type IV	Vir-like type 4 secretion system	Major pilus subunit of type IV secretion complex, VirB2
Membrane Transport	Protein and nucleoprotein secretion system, Type IV	Vir-like type 4 secretion system	Minor pilin of type IV secretion complex, VirB5
Membrane Transport	Protein and nucleoprotein secretion system, Type IV	Vir-like type 4 secretion system	Outer membrane and periplasm component of type IV secretion of T-DNA complex, has secretin-like domain, VirB9
Membrane Transport	Protein and nucleoprotein secretion system, Type IV	pVir Plasmid of Campylobacter	Hypothetical protein pVir0015
Nucleosides and Nucleotides	Pyrimidines	pyrimidine conversions	Uridine phosphorylase (EC 2.4.2.3)

**Table S4-B**	**PUCV-2 vs reference genome of *C. Jejuni* (NCTC 111168)**		
Membrane Transport	Protein secretion system, Type VI	Type VI secretion systems	Outer membrane protein ImpK/VasF, OmpA/MotB domain
Membrane Transport	Protein secretion system, Type VI	Type VI secretion systems	Uncharacterized protein ImpJ/VasE
Membrane Transport	Protein secretion system, Type VI	Type VI secretion systems	Uncharacterized protein ImpA
Membrane Transport	Protein secretion system, Type VI	Type VI secretion systems	Uncharacterized protein ImpB
Membrane Transport	Protein secretion system, Type VI	Type VI secretion systems	Uncharacterized protein ImpC
Membrane Transport	Protein secretion system, Type VI	Type VI secretion systems	Uncharacterized protein similar to VCA0109

Membrane Transport	Protein secretion system, Type VI	Type VI secretion systems	Protein ImpG/VasA
Membrane Transport	Protein secretion system, Type VI	Type VI secretion systems	Uncharacterized protein ImpH/VasB
Membrane Transport	Protein secretion system, Type VI	Type VI secretion systems	VgrG protein
Carbohydrates	Organic acids	Glycerate metabolism	Glycerate kinase (EC 2.7.1.31)
Cell Wall and Capsule	Capsular and extracellular polysacchrides	Capsular heptose biosynthesis	GDP-mannose 4,6-dehydratase (EC 4.2.1.47)
Cell Wall and Capsule	Capsular and extracellular polysacchrides	Capsular heptose biosynthesis	Capsular polysaccharide biosynthesis heptosyltransferase HddD
Phages, Prophages, Transposable elements, Plasmids	Phages, Prophages	Phage tail proteins	Phage tail length tape-measure protein
Phages, Prophages, Transposable elements, Plasmids	Phages, Prophages	Phage packaging machinery	Phage terminase, small subunit
Phages, Prophages, Transposable elements, Plasmids	Phages, Prophages	Phage packaging machinery	Phage terminase, large subunit
Phages, Prophages, Transposable elements, Plasmids	Phages, Prophages	Phage packaging machinery	Phage portal protein
Clustering-based subsystems	no subcategory	CBSS-257314.1.peg.752	Adenine-specific methyltransferase (EC 2.1.1.72)
Cell Wall and Capsule	Capsular and extracellular polysacchrides	Rhamnose containing glycans	dTDP-glucose 4,6-dehydratase (EC 4.2.1.46)
Cell Wall and Capsule	Capsular and extracellular polysacchrides	Rhamnose containing glycans	Glucose-1-phosphate thymidylyltransferase (EC 2.7.7.24)
Nucleosides and Nucleotides	Pyrimidines	pyrimidine conversions	Uridine phosphorylase (EC 2.4.2.3)

Table S4-C **PUCV-3 vs reference genome of *C. Jejuni* (NCTC 111168)**

Cell Wall and Capsule	Capsular and extracellular polysacchrides	Capsular heptose biosynthesis	Capsular polysaccharide biosynthesis heptosyltransferase HddD
Cell Wall and Capsule	Capsular and extracellular polysacchrides	Capsular heptose biosynthesis	GDP-mannose 4,6-dehydratase (EC 4.2.1.47)
DNA Metabolism	DNA replication	DNA topoisomerases, Type I, ATP-independent	DNA topoisomerase III, TraE-type (EC 5.99.1.2)
DNA Metabolism	no subcategory	Restriction-Modification System	Type III restriction-modification system DNA endonuclease res (EC 3.1.21.5)
DNA Metabolism	no subcategory	Restriction-Modification System	Type III restriction-modification system methylation subunit (EC 2.1.1.72)
Membrane Transport	Protein and nucleoprotein secretion system, Type IV	Conjugative transfer	IncQ plasmid conjugative transfer DNA nicking endonuclease TraR (pTi VirD2 homolog)
Membrane Transport	Protein and nucleoprotein secretion system, Type IV	Conjugative transfer	IncQ plasmid conjugative transfer protein TraG
Membrane Transport	Protein and nucleoprotein secretion system, Type IV	Conjugative transfer	IncQ plasmid conjugative transfer protein TraQ (RP4 TrbM homolog)
Membrane Transport	Protein and nucleoprotein secretion system, Type IV	Vir-like type 4 secretion system	ATPase required for both assembly of type IV secretion complex and secretion of T-DNA complex, VirB11
Membrane Transport	Protein and nucleoprotein secretion system, Type IV	Vir-like type 4 secretion system	ATPase required for both assembly of type IV secretion complex and secretion of T-DNA complex, VirB4
Membrane Transport	Protein and nucleoprotein secretion system, Type IV	Vir-like type 4 secretion system	Coupling protein VirD4, ATPase required for T-DNA transfer
Membrane Transport	Protein and nucleoprotein secretion system, Type IV	Vir-like type 4 secretion system	Inner membrane protein forms channel for type IV secretion of T-DNA complex, VirB3
Membrane Transport	Protein and nucleoprotein secretion system, Type IV	Vir-like type 4 secretion system	Inner membrane protein forms channel for type IV secretion of T-DNA complex, VirB8
Membrane Transport	Protein and nucleoprotein secretion system, Type IV	Vir-like type 4 secretion system	Inner membrane protein of type IV secretion of T-DNA complex, TonB-like, VirB10

Membrane Transport	Protein and nucleoprotein secretion system, Type IV	Vir-like type 4 secretion system	Inner membrane protein of type IV secretion of T-DNA complex, VirB6
Membrane Transport	Protein and nucleoprotein secretion system, Type IV	Vir-like type 4 secretion system	Lipoprotein of type IV secretion complex that spans outer membrane and periplasm, VirB7
Membrane Transport	Protein and nucleoprotein secretion system, Type IV	Vir-like type 4 secretion system	Major pilus subunit of type IV secretion complex, VirB2
Membrane Transport	Protein and nucleoprotein secretion system, Type IV	Vir-like type 4 secretion system	Minor pilin of type IV secretion complex, VirB5
Membrane Transport	Protein and nucleoprotein secretion system, Type IV	Vir-like type 4 secretion system	Outer membrane and periplasm component of type IV secretion of T-DNA complex, has secretin-like domain, VirB9
Phages, Prophages, Transposable elements, Plasmids	Phages, Prophages	Phage tail proteins	Phage tail length tape-measure protein

Table S5. Summary of prophage sequences found within genomes of *C. Jejuni* strains studied using the PHAST software

Strain	Region length Kb, completeness	First common Phage % of hit	% GC	Hits against Virus, prophage, or bacterial database or GenBank file	Most common phage name (hit_genes_count)
PUCV 1	8.0, incomplete	62.5	25.0	PHAGE_Campyl_CP220_NC_027997: hypothetical phage protein (Radical SAM family); PP_00001; phage(gi937457334)<br>PHAGE_Synech_S_SKS1_NC_020851: hypothetical protein; PP_00002; phage(gi472340890)<br>PHAGE_Synech_S_SM2_NC_015279: capsular polysaccharide biosynthesis protein; PP_00003; phage(gi326781954)<br>PHAGE_Synech_S_SKS1_NC_020851: hypothetical protein; PP_00004; phage(gi472340890)<br>putative sugar transferase [Campylobacter jejuni subsp. doylei 269.97]. gi|153951711|ref|YP_001398763.1|; PP_00005<br>PHAGE_Escher_WG01_NC_031928: hypothetical protein; PP_00006; phage(gi100045)<br>PHAGE_Shigel_SHSML_52_1_NC_031090: hypothetical protein; PP_00007; phage(gi100008)<br>PHAGE_Synech_S_SKS1_NC_020851: 3,4-dihydroxy-2-butanone 4-phosphate synthase; PP_00008; phage(gi472340887)	PHAGE_Synech_S_SKS1_NC_020851(5)<br>PHAGE_Synech_S_SM2_NC_015279(2)<br>PHAGE_Shigel_SHSML_52_1_NC_031090(2)<br>PHAGE_Acinet_Acj61_NC_014661(1)<br>PHAGE_Acinet_Ac42_NC_014660(1)<br>PHAGE_Yersin_vB_YenM_TG1_NC_028820(1)<br>PHAGE_Yersin_phiR1_RT_NC_019909(1)<br>PHAGE_Entero_IME08_NC_014260(1)<br>PHAGE_Campyl_CP220_NC_027997(1)<br>PHAGE_Ralsto_RSL1_NC_010811(1)<br>PHAGE_Synech_S_CAM9_NC_031922(1)
	7.3, incomplete	37.5	27.7	PHAGE_Pseudo_NP1_NC_031058: hypothetical protein; PP_00065; phage(gi100036)<br>cell division trigger factor [Campylobacter jejuni subsp. jejuni S3]. gi|384442543|ref|YP_005658795.1|; PP_00066<br>PHAGE_Bacill_G_NC_023719: gp257; PP_00067; phage(gi593777713)<br>PHAGE_Caulob_CcrColossus_NC_019406: putative peptide formylmethionine deformylase; PP_00068; phage(gi414088160)<br>PROPHAGE_Escher_Sakai: putative ATP-dependent protease; PP_00069; phage(gi15833954)<br>hypothetical protein A911_00915 [Campylobacter jejuni subsp. jejuni PT14]. gi|407941653|ref|YP_006857293.1|; PP_00070<br>putative kinase [Campylobacter jejuni subsp. jejuni PT14]. gi|407941652|ref|YP_006857292.1|; PP_00071<br>PHAGE_Prochl_P_SSM7_NC_015290: PRGA-formyltransferase; PP_00072; phage(gi326784531)<br>PHAGE_Bacill_CP_51_NC_025423: putative resistance protein; PP_00073; phage(gi725949539)	PHAGE_Synech_S_SKS1_NC_020851(5)<br>PHAGE_Synech_S_SM2_NC_015279(2)<br>PHAGE_Shigel_SHSML_52_1_NC_031090(2)<br>PHAGE_Acinet_Acj61_NC_014661(1)<br>PHAGE_Acinet_Ac42_NC_014660(1)<br>PHAGE_Yersin_vB_YenM_TG1_NC_028820(1)<br>PHAGE_Yersin_phiR1_RT_NC_019909(1)<br>PHAGE_Entero_IME08_NC_014260(1)<br>PHAGE_Campyl_CP220_NC_027997(1)<br>PHAGE_Ralsto_RSL1_NC_010811(1)<br>PHAGE_Synech_S_CAM9_NC_031922(1)

**PUCV 2**	8.1, incomplete	11.11	29.8	PHAGE_Synech_S_SSM7_NC_015287: putative nucleotide-sugar epimerase; PP_00207; phage(gi326783792)<br>PHAGE_Prochl_P_TIM68_NC_028955: putative nucleotidyltransferase; PP_00208; phage(gi971760366)<br>PHAGE_Caulob_Cr30_NC_025422: phosphoheptose isomerase; PP_00209; phage(gi725949171)<br>ADP-heptose--LPS heptosyltransferase II [Campylobacter jejuni subsp. jejuni PT14]. gi|407942530|ref|YP_006858173.1|; PP_00210<br>PHAGE_Staphy_phiN315_NC_004740: probable ss-1,3-N-acetylglucosaminyltransferase; PP_00211; phage(gi30043988)<br>Tgh003 [Campylobacter jejuni subsp. jejuni M1]. gi|384441768|ref|YP_005658071.1|; PP_00212<br>PHAGE_Synech_ACG_2014f_NC_026927: hypothetical protein; PP_00213; phage(gi100232)<br>PHAGE_Synech_S_CAM9_NC_031922: terminase large subunit; PP_00214; phage(gi100031)	PHAGE_Cronob_vB_CsaM_GAP32_NC_019401(1<br>) PHAGE_Prochl_P_SSM7_NC_015290(1)<br>PHAGE_Synech_S_SSM7_NC_015287(1)<br>PHAGE_Entero_vB_KleM_RaK2_NC_019526(1)<br>PHAGE_Pseudo_phiPsa374_NC_023601(1)<br>PHAGE_Bacill_Shanette_NC_028983(1)<br>PHAGE_Synech_S_SKS1_NC_020851(1)<br>PHAGE_Synech_ACG_2014f_NC_026927(1)<br>PHAGE_Caulob_Cr30_NC_025422(1)<br>PHAGE_Prochl_P_SSM2_NC_006883(1)<br>PHAGE_Vibrio_VpKK5_NC_026610(1)<br>PHAGE_Strept_Dp_1_NC_015274(1)<br>PHAGE_Rhizob_RHEph06_NC_027296(1)<br>PHAGE_Bacill_JL_NC_028982(1)<br>PHAGE_Escher_4_NC_027364(1)<br>PHAGE_Bacill_G_NC_023719(1)<br>PHAGE_Caulob_CcrColossus_NC_019406(1)<br>PHAGE_Pseudo_VCM_NC_029065(1)<br>PHAGE_Pseudo_NP1_NC_031058(1)<br>PHAGE_Bacill_CP_51_NC_025423(1)<br>PHAGE_Escher_121Q_NC_025447(1)
	10.7, incomplete	21.42	27.2	PHAGE_Synech_S_CAM7_NC_031927: phosphate-induced stress protein; PP_00217; phage(gi100227)<br>PHAGE_Staphy_phiN315_NC_004740: probable ss-1,3-N-acetylglucosaminyltransferase; PP_00218; phage(gi30043988)<br>hypothetical; PP_00219<br>putative butyryltransferase [Campylobacter jejuni 4031]. gi|549693118|ref|YP_008624076.1|; PP_00220<br>PHAGE_Aeromo_PX29_NC_023688: hypothetical protein; PP_00221; phage(gi593773872)<br>FIG01210424: hypothetical protein [Campylobacter jejuni 4031]. gi|549693116|ref|YP_008624074.1|; PP_00222<br>unknown [Campylobacter jejuni 4031]. gi|549693115|ref|YP_008624073.1|; PP_00223<br>PHAGE_Escher_phAPEC8_NC_020079: putative dTDP-glucose 4,6-dehydratase; PP_00224; phage(gi448260373)<br>PHAGE_Entero_phi92_NC_023693: Phi92_gp066; PP_00225; phage(gi726646999)<br>PHAGE_Bacill_SPbeta_NC_001884: hypothetical protein; PP_00226; phage(gi9630147)<br>lipid A biosynthesis lauroyl acyltransferase [Campylobacter jejuni subsp. jejuni PT14]. gi|407942519|ref|YP_006858161.1|; PP_00227<br>lipopolysaccharide heptosyltransferase I [Campylobacter jejuni subsp. jejuni PT14]. gi|407942518|ref|YP_006858160.1|; PP_00228<br>PHAGE_Brucel_Pr_NC_019447: putative DNA-PolB associated Exonuclease; PP_00229; phage(gi418487886)<br>PHAGE_Prochl_P_SSM2_NC_006883: nucleotide sugar epimerase; PP_00230; phage(gi61806141)	PHAGE_Synech_ACG_2014f_NC_026927(3)<br>PHAGE_Sphing_PAU_NC_019521(2)<br>PHAGE_Synech_ACG_2014f_NC_026927(2)<br>PHAGE_Entero_phi92_NC_023693(2)<br>PHAGE_Escher_phAPEC8_NC_020079(2)<br>PHAGE_Brucel_Tb_NC_019446(1)<br>PHAGE_Bacill_SP_15_NC_031245(1)<br>PHAGE_Staphy_phiN315_NC_004740(1)<br>PHAGE_Cellul_phi4:1_NC_021788(1)<br>PHAGE_Synech_S_SKS1_NC_020851(1)<br>PHAGE_Caulob_Cr30_NC_025422(1)<br>PHAGE_Prochl_P_SSM2_NC_006883(1)<br>PHAGE_Aeromo_PX29_NC_023688(1)<br>PHAGE_Bacill_SPbeta_NC_001884(1)<br>PHAGE_Synech_S_SM2_NC_015279(1)<br>PHAGE_Bacill_G_NC_023719(1)<br>PHAGE_Cellul_phi17:2_NC_021798(1)<br>PHAGE_Brucel_Pr_NC_019447(1)

<table>
<tr><td></td><td></td><td></td><td></td><td></td><td></td></tr>
<tr><td>PUCV 3</td><td>19.3, complete</td><td>19.35</td><td>31.2</td><td>PHAGE_Staphy_phiPV83_NC_002486: repressor; PP_00179; phage(gi9635682)
hypothetical protein N755_00182 [Campylobacter jejuni subsp. jejuni 00-2544]. gi|543949153|ref|YP_008541497.1|; PP_00180
PHAGE_Lister_List_36_NC_024364: hypothetical protein; PP_00181; phage(gi658307695)
hypothetical protein N755_00180 [Campylobacter jejuni subsp. jejuni 00-2544]. gi|543949151|ref|YP_008541495.1|; PP_00182
PHAGE_Clostr_phiSM101_NC_008265: putative modification methylase dpniia; PP_00183; phage(gi110804053)
phage virion morphogenesis protein [Campylobacter jejuni subsp. jejuni 00-2544]. gi|543949149|ref|YP_008541493.1|; PP_00184
PHAGE_Pseudo_PPpW_3_NC_023006: putative tail protein; PP_00185; phage(gi564292502)
hypothetical protein N135_00335 [Campylobacter jejuni subsp. jejuni 00-2425]. gi|544061971|ref|YP_008561676.1|; PP_00186
hypothetical protein N755_00175 [Campylobacter jejuni subsp. jejuni 00-2544]. gi|543949146|ref|YP_008541490.1|; PP_00187
PHAGE_Escher_vB_EcoM_ep3_NC_025430: tail protein; PP_00188; phage(gi712913215)
PROPHAGE_Xylell_Temecula1: contractile tail sheath protein; PP_00189; phage(gi28198986)
PHAGE_Campyl_CP21_NC_019507: putative tail fiber protein; PP_00190; phage(gi422935209)
PHAGE_Vibrio_qdvp001_NC_029057: tail protein; PP_00191; phage(gi985757190)
PHAGE_Campyl_CP220_NC_027997: hypothetical protein; PP_00192; phage(gi937457381)
PHAGE_Pseudo_PPpW_3_NC_023006: putative tail-collar fibre protein; PP_00193; phage(gi564292496)
PHAGE_Pseudo_PPpW_3_NC_023006: putative tail protein I; PP_00194; phage(gi564292495)
PHAGE_Pseudo_PPpW_3_NC_023006: putative baseplate J-like protein; PP_00195; phage(gi564292494)
PHAGE_Pseudo_PPpW_3_NC_023006: putative baseplate assembly protein with lysozyme; PP_00196; phage(gi564292493)
hypothetical protein CJE0235 [Campylobacter jejuni RM1221]. gi|57237246|ref|YP_178258.1|; PP_00197
PHAGE_Pseudo_PPpW_3_NC_023006: putative baseplate assembly protein V; PP_00198; phage(gi564292492)
hypothetical protein CJE0237 [Campylobacter jejuni RM1221]. gi|57237248|ref|YP_178260.1|; PP_00199
hypothetical protein CJS3_0704 [Campylobacter jejuni subsp. jejuni S3]. gi|384443029|ref|YP_005659281.1|; PP_00200
lipoprotein [Campylobacter jejuni subsp. jejuni S3]. gi|384443030|ref|YP_005659282.1|; PP_00201
hypothetical protein N755_00162 [Campylobacter jejuni subsp. jejuni 00-2544]. gi|543949133|ref|YP_008541477.1|; PP_00202
PHAGE_Entero_UAB_Phi78_NC_020414: peptidase; PP_00203; phage(gi456351073)
hypothetical protein CJE0243 [Campylobacter jejuni RM1221]. gi|57237253|ref|YP_178266.1|; PP_00204
PHAGE_Mannhe_vB_MhM_3927AP2_NC_028766: hypothetical protein; PP_00205; phage(gi971741532)
hypothetical protein N755_00158 [Campylobacter jejuni subsp. jejuni 00-2544]. gi|543949129|ref|YP_008541473.1|; PP_00206
PHAGE_Vibrio_VHML_NC_004456: ORF26; PP_00207; phage(gi27311193)
hypothetical protein N755_00156 [Campylobacter jejuni subsp. jejuni 00-2544]. gi|543949127|ref|YP_008541471.1|; PP_00208
PHAGE_Burkho_KS10_NC_011216: hypothetical protein; PP_00209; phage(gi198449267)</td><td>PHAGE_Pseudo_PPpW_3_NC_023006(6)
PHAGE_Escher_vB_EcoM_ep3_NC_025430(4)
PHAGE_Escher_vB_EcoM_ECO1230_10_NC_027995(4) PHAGE_Vibrio_VP58.5_NC_027981(4)
PHAGE_Campyl_CPX_NC_016562(3)
PHAGE_Pseudo_JBD25_NC_027992(3)
PHAGE_Vibrio_vB_VpaM_MAR_NC_019722(3)
PHAGE_Campyl_NCTC12673_NC_015464(2)
PHAGE_Campyl_CP21_NC_019507(2)
PHAGE_Haemop_SuMu_NC_019455(2)
PHAGE_Vibrio_VHML_NC_004456(2)
PHAGE_Vibrio_X29_NC_024369(2)
PHAGE_Entero_UAB_Phi78_NC_020414(1)
PHAGE_Staphy_StauST398_2_NC_021323(1)
PHAGE_Lister_LP_083_2_NC_024383(1)
PHAGE_Mannhe_vB_MhM_3927AP2_NC_028766(1) PHAGE_Ralsto_RSF1_NC_028899(1)
PHAGE_Clostr_phiSM101_NC_008265(1)
PHAGE_Halomo_phiHAP_1_NC_010342(1)
PHAGE_Staphy_phiPV83_NC_002486(1)
PHAGE_Staphy_phiETA_NC_003288(1)
PHAGE_Staphy_JS01_NC_021773(1)
PHAGE_Shewan_1/44_NC_025463(1)
PHAGE_Burkho_KS10_NC_011216(1)
PHAGE_Vibrio_VP882_NC_009016(1)
PHAGE_Pseudo_MP29_NC_011613(1)
PHAGE_Proteu_93_NC_027390(1)
PHAGE_Lister_List_36_NC_024364(1)
PHAGE_Proteu_vB_PmiP_Pm5460_NC_028916(1)
PHAGE_Ralsto_RS138_NC_029107(1)
PHAGE_Clostr_phiCDHM11_NC_029001(1)
PHAGE_Salmon_SEN4_NC_029015(1)
PHAGE_Campyl_CP220_NC_027997(1)
PHAGE_Clostr_phiCD481_1_NC_028951(1)
PHAGE_Lister_vB_LmoM_AG20_NC_020871(1)
PHAGE_Campyl_PC14_NC_031909(1)
PHAGE_Mannhe_vB_MhM_587AP1_NC_028898(1) PHAGE_Geobac_E3_NC_029073(1)
PHAGE_Rhizob_RR1_A_NC_021560(1)
PHAGE_Pectob_PP1_NC_019542(1)
PHAGE_Clostr_phiCDHM13_NC_029116(1)
PHAGE_Entero_K1_5_NC_008152(1)
PHAGE_Salmon_SEN5_NC_028701(1)
PHAGE_Lister_LP_048_NC_024359(1)
PHAGE_Mannhe_phiMHaA1_NC_008201(1)
PHAGE_Lister_LP_125_NC_021781(1)
PHAGE_Ralsto_RSL2_NC_028950(1)
PHAGE_Clostr_phiCDHM19_NC_028996(1)
PHAGE_Vibrio_qdvp001_NC_029057(1)
PHAGE_Bacter_Lily_NC_028841(1)
PHAGE_Entero_P88_NC_026014(1)
PHAGE_Staphy_StauST398_1_NC_021326(1)
PHAGE_Burkho_phiE125_NC_003309(1)
PHAGE_Campyl_CP30A_NC_018861(1)
PHAGE_Escher_pro483_NC_028943(1)</td></tr>
</table>

<table>
<tr><td></td><td></td><td></td><td></td><td></td><td></td></tr>
<tr><td></td><td>26, incomplete</td><td>15.0</td><td>31.0</td><td>attL TTTTGTTTTTTTA
PHAGE_Burkho_BcepMu_NC_005882: gp29; PP_00211; phage(gi48696939)
PHAGE_Burkho_BcepMu_NC_005882: gp30; PP_00212; phage(gi48696940)
PHAGE_Ralsto_RSY1_NC_025115: hypothetical protein; PP_00213; phage(gi691326883)
hypothetical; PP_00214
PHAGE_Salmon_Fels_2_NC_010463: tail protein; PP_00215; phage(gi169936019)
DNA-binding protein [Campylobacter jejuni RM1221]. gi|57237265|ref|YP_178278.1|; PP_00216
hypothetical; PP_00217
hypothetical protein CJE0257 [Campylobacter jejuni RM1221]. gi|57237267|ref|YP_178280.1|; PP_00218
PHAGE_Burkho_BcepMu_NC_005882: gp02; PP_00219; phage(gi48696912)
hypothetical protein CJE0259 [Campylobacter jejuni RM1221]. gi|57237269|ref|YP_178282.1|; PP_00220
hypothetical protein CJE0260 [Campylobacter jejuni RM1221]. gi|57237270|ref|YP_178283.1|; PP_00221
hypothetical protein CJE0261 [Campylobacter jejuni RM1221]. gi|57237271|ref|YP_178284.1|; PP_00222
hypothetical; PP_00223
hypothetical protein CJS3_0693 [Campylobacter jejuni subsp. jejuni S3]. gi|384443018|ref|YP_005659270.1|; PP_00224
PHAGE_Bacill_BalMu_1_NC_030945: hypothetical protein; PP_00225; phage(gi100015)
hypothetical protein CJS3_0690 [Campylobacter jejuni subsp. jejuni S3]. gi|384443015|ref|YP_005659267.1|; PP_00226
hypothetical protein CJS3_0688 [Campylobacter jejuni subsp. jejuni S3]. gi|384443013|ref|YP_005659265.1|; PP_00227
hypothetical protein CJS3_0687 [Campylobacter jejuni subsp. jejuni S3]. gi|384443012|ref|YP_005659264.1|; PP_00228
PHAGE_Haemop_SuMu_NC_019455: phage transposase; PP_00229; phage(gi418489040)
attR TTTTGTTTTTTTA
PHAGE_Ralsto_RS138_NC_029107: integrase; PP_00230; phage(gi985760913)</td><td>PHAGE_Burkho_BcepMu_NC_005882(3)
PHAGE_Rhizob_RR1_B_NC_021557(2)
PHAGE_Rhodov_RS1_NC_020866(2)
PHAGE_Burkho_phiE255_NC_009237(2)
PHAGE_Bacill_BalMu_1_NC_030945(2)
PHAGE_Vibrio_12B12_NC_021070(2)
PHAGE_Escher_vB_EcoM_ep3_NC_025430(1)
PHAGE_Ralsto_RSA1_NC_009382(1)
PHAGE_Erwini_ENT90_NC_019932(1)
PHAGE_Pseudo_vB_PaeS_PAO1_Ab30_NC_026601(1) PHAGE_Salmon_RE_2010_NC_019488(1)
PHAGE_Rhodob_RC1_NC_020839(1)
PHAGE_Mannhe_vB_MhM_3927AP2_NC_028766(1)
PHAGE_Escher_vB_EcoM_ECO1230_10_NC_027995(1) PHAGE_Ralsto_RSY1_NC_025115(1)
PHAGE_Entero_Mu_NC_000929(1)
PHAGE_Yersin_L_413C_NC_004745(1)
PHAGE_Pseudo_B3_NC_006548(1)
PHAGE_Pseudo_vB_PaeS_PM105_NC_028667(1)
PHAGE_Salmon_Fels_2_NC_010463(1)
PHAGE_Ralsto_RS138_NC_029107(1)
PHAGE_Haemop_SuMu_NC_019455(1)
PHAGE_Entero_SfMu_NC_027382(1)
PHAGE_Pseudo_JBD24_NC_020203(1)
PHAGE_Escher_pro483_NC_028943(1)</td></tr>
</table>

<table>
<tr><td rowspan="2">NCTC 11168</td><td>8.7, incomplete</td><td>25.0</td><td>27.5</td><td>PHAGE_Prochl_P_SSM2_NC_006883: nucleotide sugar epimerase; PP_01101; phage(gi61806141)
PHAGE_Brucel_Pr_NC_019447: putative DNA-PolB associated Exonuclease; PP_01102; phage(gi418487886)
heptosyltransferase I [Campylobacter jejuni subsp. jejuni NCTC 11168 = ATCC 700819]. gi|218562747|ref|YP_002344526.1|; PP_01103
lipid A biosynthesis lauroyl acyltransferase [Campylobacter jejuni subsp. jejuni NCTC 11168 = ATCC 700819]. gi|218562748|ref|YP_002344527.1|; PP_01104
PHAGE_Bacill_SPbeta_NC_001884: hypothetical protein; PP_01105; phage(gi9630147)
PHAGE_Staphy_phiN315_NC_004740: probable ss-1,3-N-acetylglucosaminyltransferase; PP_01106; phage(gi30043988)
PHAGE_Synech_ACG_2014f_NC_026927: hypothetical protein; PP_01107; phage(gi100232)
PHAGE_Staphy_phiN315_NC_004740: probable ss-1,3-N-acetylglucosaminyltransferase; PP_01108; phage(gi30043988)</td><td>PHAGE_Synech_ACG_2014f_NC_026927(2)
PHAGE_Staphy_phiN315_NC_004740(1)
PHAGE_Brucel_Tb_NC_019446(1)
PHAGE_Cellul_phi4:1_NC_021788(1)
PHAGE_Bacill_SPbeta_NC_001884(1)
PHAGE_Cellul_phi17:2_NC_021798(1)
PHAGE_Synech_S_SKS1_NC_020851(1)
PHAGE_Caulob_Cr30_NC_025422(1)
PHAGE_Brucel_Pr_NC_019447(1)
PHAGE_Prochl_P_SSM2_NC_006883(1)</td></tr>
<tr><td>6.6</td><td>37.5</td><td>30.0</td><td>PHAGE_Bacill_SP_15_NC_031245: type II N-methyl DNA methyltransferase; PP_01389; phage(gi100148)
PHAGE_Caulob_Cr30_NC_025422: phosphoheptose isomerase; PP_01390; phage(gi725949171)
PHAGE_Caulob_Cr30_NC_025422: D,D-heptose 7-phosphate kinase; PP_01391; phage(gi725949173)
methyltransferase family protein [Campylobacter jejuni subsp. jejuni NCTC 11168 = ATCC 700819]. gi|218563030|ref|YP_002344809.1|; PP_01392
PHAGE_Synech_ACG_2014f_NC_026927: hypothetical protein; PP_01393; phage(gi100233)
PHAGE_Synech_ACG_2014f_NC_026927: hypothetical protein; PP_01394; phage(gi100224)
hypothetical protein Cj1429c [Campylobacter jejuni subsp. jejuni NCTC 11168 = ATCC 700819]. gi|218563033|ref|YP_002344812.1|; PP_01395
PHAGE_Escher_phAPEC8_NC_020079: putative dTDP-4-dehydrorhamnose 3,5-epimerase; PP_01396; phage(gi448260371)</td><td>PHAGE_Synech_S_CAM7_NC_031927(3)
PHAGE_Synech_S_SKS1_NC_020851(2)
PHAGE_Entero_phi92_NC_023693(2)
PHAGE_Caulob_Cr30_NC_025422(2)
PHAGE_Escher_phAPEC8_NC_020079(2)
PHAGE_Sphing_PAU_NC_019521(2)
PHAGE_Prochl_P_SSM2_NC_006883(2)
PHAGE_Bacill_SP_15_NC_031245(1)
PHAGE_Bacill_G_NC_023719(1)
PHAGE_Synech_ACG_2014f_NC_026927(1)</td></tr>
</table>
